# Incidental and secondary findings in trio exome sequencing

**DOI:** 10.1016/j.gendis.2023.101137

**Published:** 2023-10-11

**Authors:** Camille Cohen, Emeline Bellanger, Jeremie Mortreux, Laure Raymond, François Vialard, Rodolphe Dard

**Affiliations:** a Department of Genetics, Centre Hospitalier Intercommunal de Poissy-Saint-Germain-en-Laye, Poissy 78300, France; b RHuMA, UMR-BREED, INRA-ENVA-UVSQ, Montigny le Bretonneux 78180, France; c Service de Génétique, Laboratoire Eurofins Biomnis, Lyon 69007, France

Exome sequencing (ES) generates secondary findings (SFs) in 2 % of tested individuals if one follows the American College of Medical Genetics and Genomics (ACMG) guidelines.^1^^,^^2^ However, the rate of incidental and secondary findings (ISFs) is higher in routine clinical practice because of (i) the use of trio ES instead of solo sequencing and (ii) the exclusion of the incidental findings (IFs) of medical value concerning genes in the ACMG list. Hence, it is not clear how sufficient is a restricted list of genes to detect every ISF of major clinical value; and what is the amount of additional workload for the laboratory. Using 100 trio ES datasets, we determined the proportion of pathogenic or likely-pathogenic ISFs and their clinical value (See supplementary file 1 for Materials and Methods). We evaluated the accuracy of three lists of genes (see supplementary file 2) for detection of SFs: the 2021 ACMG v3 SFs list, a list of genes involved in treatable intellectual disabilities^3^ (treat-ID list), and a list of genes involved in the 20 most frequent diseases in general populations (CS20 list).

When considering 100 trio ES datasets with prenatal and postnatal indications (Fig. 1A), we were able to establish a diagnosis in 36 cases and identified 526 P/LP variants related to SFs (Fig. 1B, C; supplementary file 3; 335 (63.7 %) P and 191 (36.3 %) LP). The median (range) number of ISFs per trio was 5 (1–13) (Fig. 1D). The 526 ISF variants were found in 400 genes. The level of redundancy was low (Fig. 1E): 314 (78.5 %) genes were reported once, 64 (16.0 %) were reported twice, and 22 (5.5 %) were reported three times or more. Of the 400 genes, 316 (79.0 %) were associated with autosomal recessive diseases and carrier status only, 54 (13.5 %) were associated with autosomal dominant diseases, 25 (6.2 %) were associated with autosomal dominant/autosomal recessive diseases, and 5 (1.2 %) were associated with X-linked diseases (Fig. 1F). It is noteworthy that we found 12 variants in 6 genes with pseudogenes (*ABCC6*, *CYP21A2*, *NCF1*, *NEB*, *SMN2*, and *TTN*).

**Figure 1:**
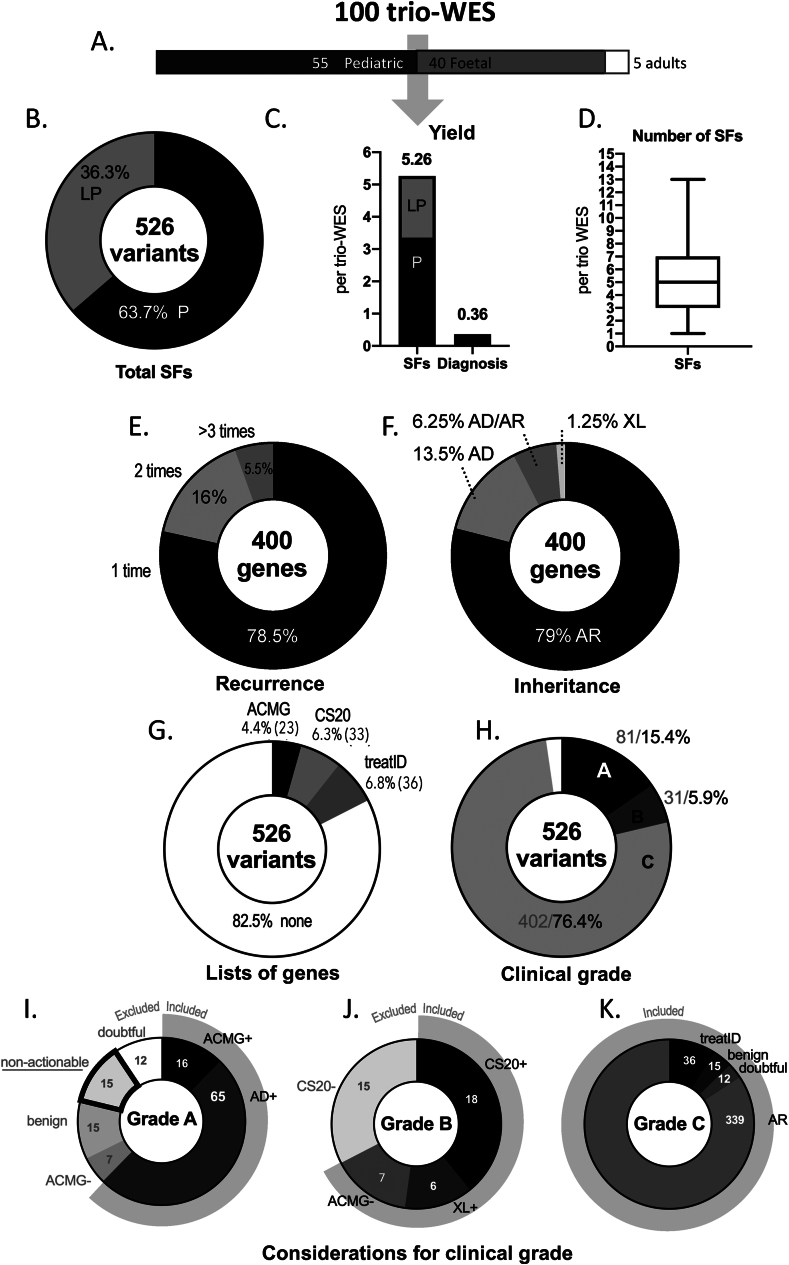
Secondary findings (SFs) in 100 trio exome sequencing (ES). **(A)** Cohort description. **(B)** Total P/LP variants as SFs per 100 trio ES. **(C)** SFs and diagnosis yield per trio ES. **(D)** Median, minimum, maximum, and quartiles of the number of SFs per trio ES. **(E)** Recurrence in genes of SFs. **(F)** mode of inheritance in genes of SFs. **(G)** SFs found in the 3 lists of genes. **(H)** Clinical grade of SFs. **(I–K)** Considerations for clinical grade, respectively for high clinical impact, familial interest and low clinical impact.

Of 526 ISFs, 81 (15.4 %) were of major clinical value for the patient (grade A; supplementary file 1; Fig. 1H, I). All are found in genes related to autosomal dominant diseases. Of the 81 ISFs, 16 variants in genes of the ACMG's list and 65 variants in other genes associated with oncologic, hemostatic, hematologic, cardiovascular, nephrologic, or ophthalmologic conditions, or inborn errors of metabolism.

Of the 49 variants that were not reportable, 15 were related to neurodegenerative or neuromuscular disorders with no current means of prevention or cure, 15 were related to benign diseases, 12 were of doubtful significance (variants related to severe malformation syndromes but detected in apparently healthy carriers), and 7 of the ACMG list did not meet the guidelines criteria.

Of the 31 (5.9 %) variants that were of great value for genetic counseling (grade B; Fig. 1H, J), 18 heterozygous pathogenic gene variants associated with autosomal recessive diseases (all present in the CS20 list), 6 variants associated with X-linked diseases, and 7 variants were from ACMG list that did not meet the guidelines criteria. Nine likely pathogenic variants (ACMG class 4) in the CS20 list were not considered for reporting because of no family history. Furthermore, 6 variants in 5 genes on the CS20 list were not considered for family screening because the corresponding pathology was not usually serious enough to justify the prenatal diagnosis and family planning (*GJB2*, *MEFV*, *F11*, and *CNGB3*) or because standard ES is unable to detect the genetic anomaly (*SMN1* deletion).

Finally, 402 (76.4 %) variants were of lower clinical value (grade C) (Fig. 1H, K). All were found in genes related to very rare autosomal recessive diseases with a heterozygous status, for which only consanguinity or family history could justify family screening. We also considered 15 variants associated with benign disease and 12 doubtful autosomal dominant variants that might prompt further phenotyping in some situations.

Of the 526 ISF variants, 92 (17.5 %) were found in one or more of the three gene lists (Fig. 1G): 23 (4.4 %) in the ACMG list, 33 (6.3 %) in the CS20 list, and 36 (6.8 %) in the treatID list. Sixteen of the 23 variants found in the ACMG list should be reported, according to the ACMG statement.

Overall, just 16 % of trio ES datasets contained SFs of the ACMG list. However, 81 % of trio ES contained ISFs of major clinical relevance for the patient, and at least 31 % for family planning. Furthermore, 15 % of trio ES contained ISFs related to severe autosomal dominant disorders that would probably affect the carrier in his/her lifetime but for which no effective treatments are available; hence, these ISFs must not be reported.

The efficiency of the lists of genes in detecting ISFs of major clinical value appears disappointing. Exhaustive analysis revealed 81 variants of great clinical value when only 16 of them have been detected by the list approach. Nevertheless, the ACMG v3 list was the only list able to detect these variants when CS20 and treatID lists failed to highlight variants of great clinical value for the patient. This yield appears to be disappointingly low and we recommend considering clinical value for the patient and family in every ISF found in ES.

Our results showed that at least one ISF was present in every trio ES dataset. Luckily, not all ISFs are of major clinical relevance, so not all ISFs have to be reported. Nevertheless, the discovery of ISFs means that the analysis takes longer and the results must be discussed more extensively by laboratory geneticists and clinicians. This situation is of great concern regarding clinical management, the laboratory's workload, patient information, and ethical aspects. It is important to bear in mind the intrinsic difference between IFs and SFs. This distinction is crucial and is highlighted by the French national guidelines.^4^ One of the common distinctions relates to the list of genes. We demonstrated that the use of a short list fails to capture all the SFs with clinical value. As already mentioned by experts, gene lists should not be used as a screening test *per se*.^5^ A holistic approach to ES data involved considering a mixture of variants of clinical relevance with a major impact independently of the phenotype. Although gene lists are of great interest in specific conditions, many ISFs are found in non-listed genes and should perhaps be reported to the patient. Acceptance and understanding by the patient are paramount.

The uncertainty of disease onset must be highlighted. The patient must understand the concept of variable expressivity, incomplete penetrance, and predisposition. The exact disease risk is always difficult to estimate. This feature of genetic ISFs sets them apart from imaging incidentalomas.

Lastly, we must also consider the large amount of variants of unknown significance hiding even more ISFs to be discovered in the years to come.

## Ethics declaration

The study was conducted anonymously with de-identified data. All patients provided their written informed consent, and the study was conducted in accordance with the Declaration of Helsinki.

## Author contributions

Camille Cohen: conceptualization, investigation, data curation; Emeline Bellanger: data curation, formal analysis, investigation; Jeremie Mortreux: data curation, methodology, validation, writing-review & editing; Laure Raymond: methodology, validation, writing-review & editing; François Vialard: conceptualization, methodology, resources, validation, writing-review & editing; Rodolphe Dard: conceptualization, data curation, formal analysis, supervision investigation, methodology, validation, writing-original draft.

## Data availability

The data are available upon request to the corresponding author.

## Conflict of interests

The authors have no conflict of interests to declare.

## Appendix A Supplementary data

Multimedia component 1 Supplementary file 1: Materials & Methods. Multimedia component 1

Multimedia component 2 Supplementary file 2: Genes lists description. Multimedia component 2

Multimedia component 3 Supplementary file 3: Detailed data of SFs found in 100 trio ES. Multimedia component 3
