# Supplementary material for: Incidental and secondary findings in trio exome sequencing
Source: Genes Dis. 2023 Oct 11;11(4):101137. doi: 10.1016/j.gendis.2023.101137 (PMC10958690; doi:10.1016/j.gendis.2023.101137)
Supplement: Multimedia component 1 — Supplementary file 1: Materials & Methods. [file mmc1.docx]

**MATERIALS & METHODS**

**Study population**

A total of 100 trio ES datasets generated in our center between September 1^st^, 2020, and September 1^st^, 2021, were included retrospectively. Other ES strategies (solo, duo, quartet etc.) were excluded. All patients had a dedicated genetic consultation for discussion of the ES results and SFs in our department and, in accordance with the French legislation and good ethical practice, gave their written, informed consent to clinical and research uses of the ES and ISF data. The indication for the ES was variously (i) a pediatric neurodevelopmental disorder and/or malformation, (ii) a fetal anomaly detected during an ongoing pregnancy or after a termination of pregnancy, (iii) intra-uterine fetal death, and (iv) disease in an adult.

**Exome sequencing**

Exome sequencing: genomic DNA was extracted using the QIAsymphony® DSP DNA Mini Kit on a QIAsymphony® instrument (QIAGEN, [Venlo](https://en.wikipedia.org/wiki/Venlo), The [Netherlands](https://en.wikipedia.org/wiki/Netherlands)), according to the manufacturer’s instructions. 50 ng of genomic DNA were included in the library preparation step. The pooled, captured libraries were generated using the Exome kit (Twist Bioscience, San Francisco, CA, USA), according to the manufacturer’s instructions. The exome panel targeted 36.8 Mb of human protein-coding genes and covered >99% of the RefSeq, CCDS, and GENCODE databases. The 36.8 Mb were covered with a design size of 41.2 Mb. The exome was sequenced on a NextSeq2000® system (Illumina, San Diego, CA, USA). Using SeqOne® software (SeqOne Genomics, Montpellier, France), sequence reads were trimmed to remove read-through adaptors and low-quality sequences. After the removal of PCR duplicates, the bases were recalibrated and variants were then called.

**Bioinformatic processing and variant sorting**

A tertiary analysis was performed with SeqOne® software (SeqOne Genomics). For each trio ES dataset, we removed technical artefacts or low mosaicism by selecting only variations with a high-quality or very-high-quality base call (Phred score >30; reliability >99.9%) and an allele frequency greater than 30% in one of the trio’s individuals. To approach clinical relevance, we then selected only rare alleles (GnomAD frequency <1% and a sample frequency <3%) in OMIM morbid genes and known or predicted to be P/LP (ACMG class 5 or 4) in ClinVar (<https://www.ncbi.nlm.nih.gov/clinvar/>) and/or VarSome (https://varsome.com/). Variants related to the suspected primary diagnosis were excluded, in order to select only variants related to SFs in the patient and/or one of the relatives.

**Lists of genes of interest**

After considering the discussions and suggestion in the literature, we defined three lists of genes of clinical interest (see supplemental S1). The first list was the ACMG list v3.0 (April 2021)^2^ of 73 highly actionable genes to be considered as SFs. Most of these genes concern cancer and cardiovascular diseases. The second list (the treatID list) included 142 genes listed by Hoytema van Konijnenburg et al. in 2021^3^. Most of these genes are related to autosomal-recessive (AR) diseases, concern inborn errors of metabolism and are subject to healthy carrier screening. The third list (the CS20 list) included the 20 most frequent AR diseases in Caucasian populations^6^. The carrier frequency in the general population was greater than 1/250, which corresponds to a risk for a pregnancy above 1/1000 (already set as threshold for prenatal invasive testing for Down’s syndrome in France).

The degree of overlap between the three lists was very small: the ACMG list and treatID list had three genes in common, the ACMG list and the CS20 list had two genes in common, and the treatID list and CS20 list had six genes in common. It is noteworthy that 14 genes in the treatID list are related to mitochondrial DNA which is not sequenced by conventional ES, as well as SMN1 exon 7 deletion in the CS20 list.

**Clinical Significance**

We evaluated the ISFs’ clinical significance by sorting them into three categories. Grade A corresponded to variants with a major clinical impact on the patient: he/she already had the disease or would certainly develop it, and means of prevention, screening and treatment were available. Hence, the patient should be told about these variants. Grade A might correspond to variants in genes in the ACMG list, variants in genes linked to autosomal dominant (AD) diseases, and homozygous or compound heterozygous variants in genes linked to AR diseases. Grade B corresponded to variants with a major clinical impact for relatives: family genetic counseling might be recommended, and the patient should be told about these variants. Grade C corresponded to a low clinical impact: genetic counseling could be considered, and in some cases (depending on the family’s medical history, ethnicity, and consanguinity), one could consider telling the patient about the variants. We also applied the ACMG criteria strictly to variants in genes in ACMG list; variants in these genes are not necessarily reported to the patient.
