# Supplementary material for: Incidental and secondary findings in trio exome sequencing
Source: Genes Dis. 2023 Oct 11;11(4):101137. doi: 10.1016/j.gendis.2023.101137 (PMC10958690; doi:10.1016/j.gendis.2023.101137)
Supplement: Multimedia component 2 — Supplementary file 2: Genes lists description. [file mmc2.pdf]

ACMGv3				CS20	treatID							
ACTA2	GLA	OTC	TGFBR2	ACADM	ABCC8	BCKDK	DNAJC12	GUSB	MOCS1	NAXE	PTPS	TMLHE
ACTC1	HFE	PALB2	TMEM127	ACADS	ABCD1	BCKDK	ECHS1	HBB	MT-CO1	NFE2L2	QDPR	TPK1
ACVRL1	HNF1A	PCSK9	TMEM43	ALDOB	ABCD4	BTD	ETFA	HIBCH	MT-CO3	NPC1	SARS1	
APC	KCNH2	PKP2	TNNI3	CFTR	ACAD9	CA5A	ETFB	HLCS	MT-CPO2	NPC2	SLC18A2	
APOB	KCNQ1	PMS2	TNNT2	CNGB3	ACAT1	CAD	ETFDH	HMGCL	MT-ND1	OTC	SLC19A3	
ATP7B	LDLR	PTEN	TP53	CPT2	AGA	CBS	ETHE1	HMGCS2	MT-ND4	PAH	SLC25A12	
BMPR1A	LMNA	RB1	TPM1	CYP21A2	AHCY	CFTR	FARSB	IARS1	MT-ND5	PCCA	SLC25A13	
BRCA1	MAX	RET	TRDN	DHCR7	ALDH5A1	COQ5	FOLR1	IDS	MT-ND6	PCCB	SLC25A15	
BRCA2	MEN1	RPE65	TSC1	DPYD	ALDH7A1	COQ8A	FUCA1	IDUA	MT-TF	PDHA1	SLC25A19	
BTD	MLH1	RYR1	TSC2	F11	AMT	CP	GAMT	IVD	MT-TH	PDHB	SLC2A1	
CACNA1S	MSH2	RYR2	TTN	GAA	AP1S1	CPS1	GATM	KCNJ11	MT-TL1	PDHX	SLC35A2	
CASQ2	MSH6	SCN5A	VHL	GBA	ARG1	CYP21A2	GCDH	LARS1	MT-TQ	PDP1	SLC35C1-CDG	
COL3A1	MUTYH	SDHAF2	WT1	GJB2	ARSA	CYP27A1	GCH1	LMBRD1	MT-TS1	PHGDH	SLC39A8	
DSC2	MYBPC3	SDHB		HBB	ASL	DBT	GLDC	MAN2B1	MT-TS2	PIGA	SLC46A1	
DSG2	MYH11	SDHC		HEXA	ASS1	DBT	GLUL	MARS1	MT-TW	PIGM	SLC5A6	
DSP	MYH7	SDHD		MEFV	ATP7A	DDC	GOT2	MFSD8	MTHFR	PIGO	SLC6A8	
ENG	MYL2	SMAD3		PAH	ATP7B	DHCR7	GRIN1	MMAA	MTHFS	PMM2	SPR	
FBN1	MYL3	SMAD4		PMM2	BCKDHA	DHFR	GRIN2A	MMAB	MTR	PNPO	TAT	
FLNC	PRKAG2	STK11		PYGM	BCKDHB	DLAT	GRIN2B	MMACHC	MTRR	PRPS1	TCN2	
GAA	NF2	TGFBR1		SMN1	BCKDHB	DLD	GRIN2D	MMUT	NAGS	PSPH	TH	

	ACMGv3	CS20	treatID
ACMGv3	73	2	3
CS20		20	6
treatID			142
