# Supplementary material for: Incidental and secondary findings in trio exome sequencing
Source: Genes Dis. 2023 Oct 11;11(4):101137. doi: 10.1016/j.gendis.2023.101137 (PMC10958690; doi:10.1016/j.gendis.2023.101137)
Supplement: Multimedia component 3 — Supplementary file 3: Detailed data of SFs found in 100 trio ES. [file mmc3.pdf]

Case	Genes lists				ACMG				
	ACMG	CS20	treatID						
1				MME	4	NM_000902.5:c.1579C>T	NP_000893.2:p.Arg527Ter	Charcot-Marie-Tooth disease	AD,AR
				BBS1	4	NM_024649.5:c.479G>A	NP_078925.3:p.Arg160Gln	Bardet-Biedl	AR
			X	HMGCL	4	NM_000191.3:c.3G>T	NP_000182.2:p.Met1?	Deficiency of hydroxymethylglutaryl-CoA lyase	AR
				SLC5A2	5	NM_003041.4:c.384C>G	NP_003032.1:p.Tyr128Ter	Familial renal glucosuria	AD,AR
				SLC10A2	5	NM_000452.3:c.556del	NP_000443.2:p.Trp186GlyfsTer23	Bile acid malabsorption	AR
2				KIZ	4	NM_018474.6:c.1675+2T>C		Retinitis pigmentosa	AR
				MED25	4	NM_030973.4:c.1556del	NP_112235.2:p.Lys519ArgfsTer78	Basel-Vanagaite-Smirin-Yosef syndrome	AR
				NDUFA11	4	NM_001193375.3:c.470_471del	NP_001180304.1:p.Ser157TrpfsTer270	Mitochondrial complex 1 deficiency	AR
				STRC	4	NM_153700.2:c.3460C>T	NP_714544.1:p.Arg1154Ter	Deafness	AR
				TBC1D7	4	NM_001318806.2:c.395G>A	NP_001305735.1:p.Trp132Ter	Macrocephaly/megalencephaly syndrome	AR
				MTMR2	4	NM_016156:c.81_82insTCMAR-TIGGER	NM_016156:p.Leu27_Pro28insTCMAR-TIGGER	Charcot-Marie-Tooth disease	AR
				SNIP1	4	NM_024700.4:c.331C>T	NP_078976.2:p.Arg111Cys	Psychomotor retardation, epilepsy, and craniofacial dysmorphism	AR
				ACSF3	4	NM_174917.5:c.593T>G	NP_777577.2:p.Met198Arg	Combined malonic and methylmalonic aciduria	AR
				EYS	5	NM_001142800.2:c.2981del	NP_001136272.1:p.Pro994LeufsTer10	Retinitis pigmentosa	AR
				DST	5	NM_015548.5:c.15129+1G>T		Neuropathy, hereditary sensory and autonomic	AR
				IL17RD	5	NM_017563.5:c.676G>A	NP_060033.3:p.Gly226Ser	Hypogonadotropic hypogonadism	AR
3	X			TTN	4	NM_003319.4:c.35527C>T	NP_003310.4:p.Arg11843Ter	Familial hypertrophic cardiomyopathy	AD
				CAPN3	4	NM_000070.3:c.1250C>T	NP_000061.1:p.Thr417Met	Limb-girdle muscular dystrophy	AD,AR
				GAS2L2	4	NM_139285.4:c.887_890del	NP_644814.1:p.Val296GlyfsTer13	Ciliary dyskinesia, primary,	AR
				CYP24A1	5	NM_000782.5:c.1493C>G	NP_000773.2:p.Ser498Ter	Hypercalcemia,	AR
				SLC29A3	5	NM_018344.6:c.73C>T	NP_060814.4:p.Arg25Ter	Histiocytosis-lymphadenopathy plus syndrome	AR
				OPLAH	5	NM_017570.5:c.2142_2143del	NP_060040.1:p.Asp716HisfsTer34	5-Oxoprolinase deficiency	AD,AR
				METTL23	5	NM_001080510.5:c.169_172del	NP_001073979.3:p.His57ValfsTer11	Mental retardation,	AR
				ACY1	5	NM_000666.3:c.699A>C	NP_000657.1:p.Glu233Asp	Aminoacylase 1 deficiency	AR
				TMEM126B	5	NM_018480.7:c.397G>A	NP_060950.3:p.Asp133Asn	Mitochondrial complex 1 deficiency	AR
				VWF	5	NM_000552.5:c.2561G>A	NP_000543.3:p.Arg854Gln	von Willebrand disease	AD
4				PNKP	4	NM_007254.4:c.1029+2T>C		Early infantile epileptic encephalopathy	AR
				KIAA0586	4	NM_014749.5:c.392del	NP_055564.3:p.Arg131LysfsTer4	Joubert syndrome	AR
				TSPYL1	4	NM_003309.4:c.725_726del	NP_003300.1:p.Val242GlufsTer52	Sudden infant death with dysgenesis of the testes syndrome	AR
				GRID2	5	NM_001510.4:c.670C>T	NP_001501.2:p.Arg224Ter	Spinocerebellar ataxia	AR
				MYO15A	5	NM_016239.4:c.7124_7127del	NP_057323.3:p.Asp2375ValfsTer41	Deafness	AR
5				COL4A3	4	NM_000091.5:c.4981C>T	NP_000082.2:p.Arg1661Cys	Alport syndrome	AD
				GALT	4	NM_000155.4:c.502G>A	NP_000146.2:p.Val168Met	Deficiency of UDPglucose-hexose-1-phosphate uridylyltransferase	AR
				PHYH	4	NM_006214.4:c.512G>A	NP_006205.1:p.Arg171His	Phytanic acid storage disease	AR
				MPV17	5	NM_002437.5:c.148C>T	NP_002428.1:p.Arg50Trp	Navajo neurohepatopathy	AR
				AEBP1	5	NM_001129.5:c.2925del	NP_001120.3:p.Thr976LeufsTer21	Ehlers-danlos syndrome	AR
				SKIV2L	5	NM_006929.5:c.3187C>T	NP_008860.4:p.Arg1063Ter	Trichohepatoenteric syndrome	AR
6	X			TTN	4	NM_003319.4:c.71104del	NP_003310.4:p.Arg23702GlyfsTer26	Familial hypertrophic cardiomyopathy	AD
				MARVELD2	4	NM_001038603.3:c.1583_1601del	NP_001033692.2:p.Arg528LeufsTer6	Deafness	AR
				SLC12A6	4	NM_005135.2:c.965+1G>A		Agenesis of the corpus callosum with peripheral neuropathy	AR
				SNIP1	4	NM_024700.4:c.331C>T	NP_078976.2:p.Arg111Cys	Psychomotor retardation, epilepsy, and craniofacial dysmorphism	AR
				FLG	5	NM_002016.2:c.3837del	NP_002007.1:p.Ser1280GlnfsTer166	Ichthyosis vulgaris	AD
				UBAP1	5	NM_001171201.1:c.75C>A	NP_001164672.1:p.Tyr25Ter	Spastic paraplegia	AD
				SLC25A26	5	NM_173471.4:c.33+1G>A		Combined oxidative phosphorylation deficiency	AR
7				PNPLA6	5	NM_001166111.2:c.28-1G>A		Spastic paraplegia	AR
				CD46	5	NM_002389.4:c.944C>G	NP_002380.3:p.Ser315Ter	Atypical hemolytic-uremic syndrome	AD,AR
8				GPR179	4	NM_001004334.4:c.6433C>T	NP_001004334.3:p.Gln2145Ter	Congenital stationary night blindness	AR
				MYSM1	5	NM_001085487.3:c.695T>A	NP_001078956.1:p.Leu232Ter	Bone marrow failure syndrome 4	AR
				BBS9	5	NM_014451.4:c.670C>T	NP_055266.2:p.Gln224Ter	Bardet-Biedl syndrome 9	AR
9				COG1	4	NM_018714.3:c.1680C>G	NP_061184.1:p.Tyr560Ter	congenital disorder of glycosylation	AR
				SH2B3	4	NM_005475.3:c.1183G>A	NP_005466.1:p.Glu395Lys	Thrombocythemia	AD
				GALT	5	NM_000155.4:c.563A>G	NP_000146.2:p.Gln188Arg	Deficiency of UDPglucose-hexose-1-phosphate uridylyltransferase	AR

				RAD50	5	NM_005732.4:c.1969+1G>C		Nijmegen breakage syndrome	AR
				FLG	5	NM_002016.2:c.7339C>T	NP_002007.1:p.Arg2447Ter	Ichthyosis vulgaris	AD
10				ANO1	4	NM_018075.5:c.289del	NP_060545.3:p.Met97Ter	Spinocerebellar ataxia	AR
				RAPSN	5	NM_005055.5:c.43_52del	NP_005046.2:p.Gln15SerfsTer46	Myasthenic syndrome, congenital	AR
				GPR179	5	NM_001004334.4:c.2683C>T	NP_001004334.3:p.Arg895Ter	Congenital stationary night blindness	AR
				ARMC9	5	NM_025139.6:c.178-1G>A		Joubert syndrome	AR
11				TMEM260	4	NM_017799.4:c.2087_2091del	NP_060269.3:p.Lys696ThrfsTer7	Structural heart defects and renal anomalies syndrome	AR
				MKS1	4	NM_017777.4:c.1408-2del		Meckel syndrome	AR
				CNGA3	4	NM_001298.3:c.847C>T	NP_001289.1:p.Arg283Trp	Achromatopsia	AR
	X		X	ATP7B	4	NM_000053.4:c.122A>G	NP_000044.2:p.Asn41Ser	Wilson disease	AR
				CEP290	5	NM_025114.4:c.6424del	NP_079390.3:p.Glu2142LysfsTer10	Meckel syndrome	AR
				FTCD	5	NM_006657.3:c.1607T>A	NP_006648.1:p.Leu536Ter	Glutamate formiminotransferase deficiencency	AR
12				FANCM	4	NM_020937.4:c.5791C>T	NP_065988.1:p.Arg1931Ter	Fanconi anemia	AR
				MYO15A	5	NM_016239.4:c.6638G>A	NP_057323.3:p.Trp2213Ter	Deafness	AR
				ATIC	5	NM_004044.7:c.923-2A>G		Deafness	AR
				USH1C	5	NM_005709.4:c.238dup	NP_005700.2:p.Arg80ProfsTer69	Usher syndrome, type 1C	AR
				CRB2	5	NM_173689.7:c.2445_2446del	NP_775960.4:p.Cys816PhefsTer12	Ventriculomegaly with cystic kidney disease	AR
				UBA5	5	NM_024818.6:c.1111G>A	NP_079094.1:p.Ala371Thr	Epileptic encephalopathy	AR
				PDE6C	5	NM_006204.4:c.1300_1301del	NP_006195.3:p.Leu434LysfsTer3	Cone dystrophy	AR
				FYCO1	5	NM_024513.4:c.101_102dup	NP_078789.2:p.Ile35ProfsTer48	Cataract	AR
				TYMP	5	NM_001953.5:c.866A>C	NP_001944.1:p.Glu289Ala	Mitochondrial DNA depletion syndrome	AR
				OSGEP	5	NM_017807.4:c.328T>C	NP_060277.1:p.Cys110Arg	Galloway-Mowat syndrome	AR
13		X		HEXA	4	NM_000520.6:c.1178G>A	NP_000511.2:p.Arg393Gln	Tay-Sachs	AR
				MSH3	5	NM_002439.5:c.1448dup	NP_002430.3:p.Lys484GlnfsTer11	Familial adenomatous polyposis	AR
				ALG2	5	NM_024105.4:c.148C>T	NP_077010.1:p.Gln50Ter	congenital disorder of glycosylation	AR
14		X		ACADS	4	NM_000017.4:c.529T>C	NP_000008.1:p.Trp177Arg	Deficiency of butyryl-CoA dehydrogenase	AR
				COG5	4	NM_006348.3:c.2548C>T	NP_006339.3:p.Gln850Ter	Congenital disorder of glycosylation type 2i	AR
				KLK4	5	NM_004917.4:c.458G>A	NP_004908.4:p.Trp153Ter	Amelogenesis imperfecta, hypomaturation type, IIA1	AR
				POLR3A	5	NM_007055.4:c.3991G>A	NP_008986.2:p.Ala1331Thr	Hypomyelinating leukodystrophy	AR
15				SLC26A2	4	NM_000112.4:c.961del	p.L321SfsTer21	achondrogenesis, dysplasia	AR
				RNASEH2B	4	NM_024570.4:c.529G>A	NP_078846.2:p.Ala177Thr	Aicardi Goutieres	AR
				GNRHR	4	NM_000406.3:c.317A>G	NP_000397.1:p.Gln106Arg	Idiopathic hypogonadotropic hypogonadism	AR
				SORD	4	NM_003104.6:c.757del	NP_003095.2:p.Ala253GlnfsTer27	Sorbitol dehydrogenase deficiency	AR
				FLG	4	NM_002016.2:c.1501C>T	NP_002007.1:p.Arg501Ter	Ichthyosis vulgaris	AD
		X	X	CYP21A2	5	NM_000500.9:c.1360C>T	p.P454S	Classic congenital adrenal hyperplasia	AR
				CLCN1	5	NM_000083.3:c.1453A>G	NP_000074.3:p.Met485Val	Congenital myotonia	AD,AR
				ABCA12	5	NM_015657.3:c.2876-2A>G		Ichthyosis	AD,AR
				STAT5B	5	NM_012448.4:c.1267C>T	NP_036580.2:p.Arg423Ter	Growth hormone insensitivity with immunodeficiency	AR
				PIGN	5	NM_012327.6:c.181G>T	NP_036459.1:p.Glu61Ter	Multiple congenital anomalies-hypotonia-seizures syndrome	AR
16			X	GCDH	4	NM_000159.4:c.937C>T	NP_000150.1:p.Arg313Trp	Glutaric aciduria	AR
	X			MUTYH	4	NM_012222.2:c.1178G>A	NP_036354.1:p.Gly393Asp	Polyposis	AD,AR
				KIAA0586	4	NM_014749.5:c.392del	NP_055564.3:p.Arg131LysfsTer4	Joubert syndrome	AR
				USH2A	5	NM_007123.5:c.2276G>T	NP_009054.5:p.Cys759Phe	Usher syndrome	AR
				NCF2	5	NM_000433.3:c.904del	NP_000424.2:p.His302ThrfsTer43	Chronic granulomatous disease	AR
17				ADGRV1	4	NM_032119.4:c.6901C>T	NP_115495.3:p.Gln2301Ter	Febrile seizures	AD
		X		GJB2	4	NM_004004.6:c.101T>C	NP_003995.2:p.Met34Thr	Deafness	AR
				LRTOMT	4	NM_001205138.3:c.3G>A	NP_001192067.1:p.Met1?	Deafness	AR
		X		HEXA	4	NM_000520.6:c.1073+1G>A		Tay-Sachs	AR
				MYO7A	4	NM_000260.4:c.5101C>T	NP_000251.3:p.Arg1701Ter	Usher syndrome	AR
				NEK8	5	NM_178170.3:c.214G>T	NP_835464.1:p.Glu72Ter	renal-hepatic-pancreatic dysplasia	AR
18				VWF	4	NM_000552.4:c.2120delinsTT	NP_000543.2:p.Cys707PhefsTer6	von Willebrand disease	AD
				MID1	4	NM_000381.4:c.193dup	NP_000372.1:p.Leu65ProfsTer43	Opitz GBBB	XLR
				PRODH	4	NM_016335.5:c.1322T>C	NP_057419.5:p.Leu441Pro	Proline dehydrogenase deficiency	AR
				CEP63	4	NM_025180.4:c.1000dup	NP_079456.2:p.Ser334PhefsTer9	Seckel syndrome	AR

				BRAT1	4	NM_152743.4:c.294dup	NP_689956.2:p.Leu99ThrfsTer92	Rigidity and multifocal seizure syndrome	AR
19				G6PD	4	NM_000402.4:c.934G>C	NP_000393.4:p.Asp312His	Glucose 6 phosphate dehydrogenase deficiency	XLR
				RARS2	5	NM_020320.5:c.1A>T	NP_064716.2:p.Met1?	Pontocerebellar hypoplasia	AR
		X	X	HBB	5	NM_000518.5:c.20del	NP_000509.1:p.Glu7GlyfsTer13	Beta-thalassemia	AR
	X			TGFBR1	5	NM_004612.4:c.812del	NP_004603.1:p.Gly271ValfsTer22	Loeys Dietz Aortic Aneurysm Syndrome	AD
				NIN	5	NM_020921.3:c.3787C>T	NP_065972.3:p.Gln1263Ter	Seckel syndrome	AR
				DOCK7	5	NM_033407.3:c.4191_4194del	NP_212132.2:p.Arg1398AlafsTer13	Epileptic encephalopathy	AR
				CNTNAP1	5	NM_003632.3:c.2355G>A	NP_003623.1:p.Trp785Ter	Lethal congenital contracture syndrome	AR
20				HSD17B4	4	NM_000414.4:c.1369A>T	NP_000405.1:p.Asn457Tyr	gonadal dysgenesis	AR
				DUOX2	4	NM_014080.4:c.2895_2898del	NP_054799.4:p.Phe966SerfsTer29	Thyroid dyshormonogenesis	AR
	X			PKP2	5	NM_004572.3:c.148_151del	NP_004563.2:p.Thr50SerfsTer61	Arrhythmogenic right ventricular cardiomyopathy	AD
			X	HLCS	5	NM_000411.8:c.1180-2A>G		Holocarboxylase synthetase deficiency	AR
	X		X	BTD	5	NM_001281723.3:c.38_44delinsTCC	NP_001268652.2:p.Cys13PhefsTer36	Biotinidase deficiency	AR
				CHST3	5	NM_004273.5:c.1318_1336del	NP_004264.2:p.Val440LeufsTer72	Spondyloepiphyseal dysplasia	AR
21				DCLRE1C	4	NM_022487.4:c.1705_1708del	NP_071932.2:p.Lys569GlufsTer6	Omenn syndrome	AR
				TRIOBP	4	NM_138632.2:c.1247del	NP_619538.2:p.Cys416LeufsTer22	Deafness,	AR
				LZTR1	5	NM_006767.4:c.1256del	NP_006758.2:p.Phe419SerfsTer42	Noonan syndrome	AR
				KIAA0753	5	NM_014804.3:c.184del	NP_055619.2:p.Leu62Ter	Orofaciodigital syndrome	AR
22				FLG	4	NM_002016.2:c.9947C>G	NP_002007.1:p.Ser3316Ter	Ichthyosis vulgaris	AD
			X	MAN2B1	5	NM_000528.4:c.2923+1G>C		Deficiency of alpha-mannosidase	AR
				PI4KA	5	NM_058004.4:c.5821C>T	NP_477352.3:p.Arg1941Ter	Polymicrogyria	AR
				PGAM2	5	NM_000290.4:c.233G>A	NP_000281.2:p.Trp78Ter	Glycogen storage disease	AR
23				MUT	5	NM_000255.4:c.312del	NP_000246.2:p.Trp105GlyfsTer75	Methylmalonic aciduria	AR
				PNPLA6	5	NM_006702.5:c.2144_2146del	NP_006693.3:p.Ala715del	Spastic paraplegia	AR
24				CNGB1	4	NM_001297.4:c.2544dup	NP_001288.3:p.Leu849AlafsTer3	Retinis pigmentosa	AR
		X		GJB2	5	NM_004004.5:c.35del	NP_003995.2:p.Gly12ValfsTer2	Deafness	AR
		X	X	PMM2	5	NM_000303.2:c.422G>A	NP_000294.1:p.Arg141His	Carbohydrate-deficient glycoprotein syndrome	AR
				MCCC1	5	NM_020166.3:c.1869+1G>A		3-Methylcrotonyl-CoA carboxylase 1 deficiency	AR
25				EVC2	5	NM_147127.5:c.3100C>T	NP_667338.3:p.Gln1034Ter	Ellis van-Creveld syndrome	AR
				TMEM237	5	NM_152388.4:c.877C>T	NP_689601.2:p.Arg293Ter	Joubert syndrome	AR
		X	X	CYP21A2	5	NM_000500.9:c.844G>T	NP_000491.4:p.Val282Leu	Classic congenital adrenal hyperplasia	AR
26				TRNT1	5	NM_182916.3:c.1252dup	NP_886552.3:p.Ser418LysfsTer9	Retinitis pigmentosa and erythrocytic microcytosis	AR
27				DMGDH	4	NM_013391.3:c.326A>G	NP_037523.2:p.His109Arg	Dimethylglycine dehydrogenase deficiency	AR
				CLDN1	5	NM_021101.5:c.200_201del	NP_066924.1:p.Phe67Ter	Ichthyosis	AR
				MYO7A	5	NM_000260.4:c.655_660del	NP_000251.3:p.Ile219_His220del	Usher syndrome	AR
				AURKC	5	NM_003160.3:c.43del	NP_003151.2:p.Leu15TrpfsTer23	Infertility associated with multi-tailed spermatozoa and excessive DNA	AR
28				WDR73	4	NM_032856.4:c.1132dup	NP_116245.2:p.Arg378ProfsTer12	Galloway-Mowat syndrome	AR
				SLC3A1	4	NM_000341.4:c.592del	NP_000332.2:p.Ala198GlnfsTer8	Cystinuria	AR
				SCN1A	5	NM_006920.6:c.5113T>A	NP_008851.3:p.Cys1705Ser	Familial hemiplegic migraine	AD
				SLC12A6	5	NM_005135.2:c.1246_1252del	NP_005126.1:p.Asp416Ter	Agenesis of the corpus callosum with peripheral neuropathy	AR
				CEP164	5	NM_014956.5:c.688-2A>G		Nephronophthisis	AR
				ERCC4	5	NM_005236.3:c.579G>A	NP_005227.1:p.Trp193Ter	Xeroderma pigmentosum	AR
				NDUFA6	5	NM_002490.6:c.281_284del	NP_002481.3:p.Ile94LysfsTer44	Mitochondrial complex 1 deficiency,	AR
29				BSCL2	4	NM_032667.6:c.-105-2A>G		Spastic paraplegia	AD
				F7	4	NM_000131.4:c.847C>T	NP_000122.1:p.Arg283Trp	Factor VII deficiency	AR
				CLDN14	4	NM_012130.4:c.301G>A	NP_036262.1:p.Gly101Arg	Deafness	AR
				ABCB6	4	NM_005689.4:c.2168G>A	NP_005680.1:p.Arg723Gln	Pseudohyperkalemia	AD
				PLD1	5	NM_002662.5:c.472C>T	NP_002653.1:p.Arg158Ter	Cardiac valvular defect,	AR
				METTL23	5	NM_001080510.4:c.174_177del	NP_001073979.3:p.Cys58TrpfsTer10	Mental retardation	AR
30				SYNE1	5	NM_033071.3:c.14971_14974dup	NP_149062.1:p.Ile4992ThrfsTer54	Arthrogryposis multiplex congenita	AR
				ACSF3	5	NM_174917.5:c.1412G>A	NP_777577.2:p.Arg471Gln	Combined malonic and methylmalonic aciduria	AR
				CABP2	5	NM_016366.3:c.637+1G>T		Deafness	AR
31				PADI6	4	NM_207421.4:c.1846del	NP_997304.3:p.Asp616ThrfsTer5	Preimplantation embryonic lethality	AR
				CEP78	4	NM_032171.3:c.1801-1G>C		Cone-rod dystrophy and hearing loss	AR
				GAS2L2	4	NM_139285.4:c.887_890del	NP_644814.1:p.Val296GlyfsTer13	Ciliary dyskinesia, primary,	AR
				KCTD7	4	NM_153033.4:c.551G>A	NP_694578.1:p.Arg184His	Epilepsy, progressive myoclonic	AR

32				MKRN3	4	NM_005664.4:c.155del	NP_005655.1:p.Ala52GlufsTer9	Precocious puberty, central,	AD
				CFAP65	4	NM_194302.4:c.2443dup	NP_919278.2:p.Gln815ProfsTer30	Spermatogenic failure	AR
				KIAA0586	4	NM_014749.5:c.392del	NP_055564.3:p.Arg131LysfsTer4	Joubert syndrome	AR
				MEF2A	4	NM_005587.4:c.836C>T	NP_005578.2:p.Pro279Leu	Coronary artery disease	AD
			X	DLD	5	NM_000108.5:c.681del	NP_000099.2:p.Glu227AspfsTer13	Deficiency of cytochrome-b5 reductase	AR
				SUCLA2	5	NM_003850.3:c.881G>A	NP_003841.1:p.Trp294Ter	Mitochondrial DNA depletion syndrome	AR
				SECISBP2	5	NM_024077.5:c.880+1G>A		Thyroid hormone metabolism,	AR
	X		X	BTD	5	NM_001281723.3:c.873del	NP_001268652.2:p.Ser291ArgfsTer23	Biotinidase deficiency	AR
				DGKE	5	NM_003647.3:c.966G>A	NP_003638.1:p.Trp322Ter	Nephrotic syndrome	AR
33				C4orf26	4	NM_178497.5:c.54_56delinsTACTGGTA	NP_848592.2:p.Val20GlyfsTer145	Amelogenesis imperfecta	AR
				TRAPPC2	4	NM_001128835.3:c.12G>A	NP_001122307.2:p.Trp4Ter	Spondyloepiphyseal dysplasia tarda	XLR
				COL6A3	4	NM_004369.4:c.476dup	NP_004360.2:p.Leu160SerfsTer7	Ullrich Congenital Muscular Dystrophy	AR
				POR	4	NM_000941.3:c.859G>C	NP_000932.3:p.Ala287Pro	Antley-Bixler syndrome	AR
				ADSSL1	4	NM_152328.5:c.741del	NP_689541.1:p.Lys248ArgfsTer23	Myopathy	AR
				POLG	5	NM_002693.3:c.2243G>C	NP_002684.1:p.Trp748Ser	Mitochondrial DNA depletion syndrome	AR
		X		ACADM	5	NM_000016.6:c.985A>G	NP_000007.1:p.Lys329Glu	Medium-chain acyl-coenzyme A dehydrogenase deficiency	AR
34				FSIP2	4	NM_173651.4:c.20509del	NP_775922.3:p.Ser6837ValfsTer41	Spermatogenic failure	AR
	X			DSC2	4	NM_004949.5:c.1075del	NP_004940.1:p.Ser359LeufsTer3	Arrhythmogenic right ventricular cardiomyopathy,	AD
				KIAA0556	4	NM_015202.5:c.351del	NP_056017.4:p.Leu118Ter	Joubert syndrome	AR
				FLG	4	NM_002016.2:c.9947C>G	NP_002007.1:p.Ser3316Ter	Ichthyosis vulgaris	AD
				UBE3B	5	NM_130466.4:c.1622+1G>C		Kaufman oculocerebrofacial syndrome	AR
				ABCG8	5	NM_022437.3:c.1083G>A	NP_071882.1:p.Trp361Ter	Sitosterolemia	AR
				NSUN3	5	NM_022072.5:c.295C>T	NP_071355.1:p.Arg99Ter	Combined oxidative phosphorylation deficiency	AR
		X	X	DHCR7	5	NM_001360.3:c.964-1G>C		Smith-Lemli-Opitz syndrome	AR
35				COL4A1	4	NM_001845.6:c.626G>A	NP_001836.3:p.Gly209Asp	Microangiopathy and leukoencephalopathy	AD
				FOXE3	4	NM_012186.3:c.410G>A	NP_036318.1:p.Gly137Asp	Aortic aneurysm	AD
				EPG5	5	NM_020964.3:c.6232C>T	NP_066015.2:p.Arg2078Ter	Vici syndrome	AR
				EDAR	5	NM_022336.4:c.529+1G>A		Ectodermal dysplasia	AR
				MCPH1	5	NM_024596.5:c.1300C>T	NP_078872.3:p.Gln434Ter	Primary autosomal recessive microcephaly	AR
		X	X	CFTR	5	NM_000492.4:c.350G>A	NP_000483.3:p.Arg117His	CFTR-RD	AR
				SYCE1	5	NM_130784.4:c.163+1G>A		Spermatogenic/Premature ovarian failure	AR
				ADAMTSL4	5	NM_019032.6:c.2917C>T	NP_061905.2:p.Arg973Ter	Ectopia lentis	AR
36				SLC7A9	4	NM_014270.5:c.313G>A	NP_055085.1:p.Gly105Arg	Cystinuria	AR
				LAMA3	4	NM_001302996.2:c.1333C>T	NP_001289925.1:p.Gln445Ter	Junctional epidermolysis bullosa	AR
				CLCN1	4	NM_000083.3:c.2680C>T	NP_000074.3:p.Arg894Ter	Congenital myotonia	AR
				PKD2	5	NM_000297.4:c.1094+1G>A		Polycystic kidney disease	AD
		X	X	PAH	5	NM_000277.3:c.814G>T	NP_000268.1:p.Gly272Ter	phenylketonuria	AR
				MFRP	5	NM_031433.4:c.1150dup	NP_113621.1:p.His384ProfsTer8	Nanophthalmos	AR
37				NFIB	4	NM_001190738.2:c.2T>C	NP_001177667.1:p.Met1?	Macrocephaly, acquired, with impaired intellectual development	AD
				DNAI2	4	NM_023036.6:c.1723-1G>T		Ciliary dyskinesia, primary,	AR
				B4GALNT1	4	NM_001276469.2:c.965del	NP_001263398.1:p.Gly322AlafsTer29	Hereditary spastic paraplegia	AR
				DNAAF2	5	NM_018139.3:c.388G>T	NP_060609.2:p.Glu130Ter	Ciliary dyskinesia, primary	AR
				PROM1	5	NM_006017.3:c.276+1G>A		Stargardt disease 4	AD
				GRM1	5	NM_001278064.2:c.26dup	NP_001264993.1:p.Ala11SerfsTer78	Spinocerebellar ataxia	AD,AR
				RIPK1	5	NM_003804.6:c.1934C>T	NP_003795.2:p.Thr645Met	Autoinflammation with episodic fever and lymphadenopathy	AD,AR
38				REN	4	NM_000537.4:c.516_517del	NP_000528.1:p.Gln172HisfsTer19	Hyperuricemic nephropathy, familial juvenile	AD
				OPLAH	5	NM_017570.5:c.3457_3458del	NP_060040.1:p.Ser1153ProfsTer35	5-Oxoprolinase deficiency	AD,AR
				ABCB11	5	NM_003742.4:c.1139del	NP_003733.2:p.Leu380TrpfsTer18	familial cholestasis	AR
				DOCK3	5	NM_004947.5:c.1175G>A	NP_004938.1:p.Arg392Gln	Neurodevelopmental disorder with impaired intellectual development, hyp	AR
				ABCA4	5	NM_000350.3:c.3113C>T	NP_000341.2:p.Ala1038Val	Retinitis pigmentosa	AR
39				NTRK1	5	NM_002529.4:c.2T>A	NP_002520.2:p.Met1?	Hereditary insensitivity to pain with anhidrosis	AR
				FLG	5	NM_002016.2:c.5840G>A	NP_002007.1:p.Trp1947Ter	Ichthyosis vulgaris	AD
40				AK7	4	NM_152327.5:c.2018T>C	NP_689540.2:p.Leu673Pro	Spermatogenic failure	AR
		X		CNGB3	5	NM_019098.5:c.1148del	NP_061971.3:p.Thr383IlefsTer13	Achromatopsia	AR
				DCLRE1C	5	NM_022487.4:c.1330_1334del	NP_071932.2:p.Leu444IlefsTer17	Omenn syndrome	AR
				INSR	5	NM_000208.4:c.1431_1437delinsCC	NP_000199.2:p.Gln479GlufsTer16	Leprechaunism syndrome	AR
				FSHR	5	NM_000145.4:c.528G>A	NP_000136.2:p.Trp176Ter	Ovarian dysgenesis 1	AR

				BBS1	5	NM_024649.5:c.48-1G>A		Bardet Biedl	AR
	X			BRCA1	5	NM_007294.4:c.1059G>A	NP_009225.1:p.Trp353Ter	Breast-ovarian cancer	AR
41				ARMC9	4	NM_001271466.4:c.2201delinsTG	NP_001258395.2:p.Thr734MetfsTer233	Joubert syndrome	AR
				SCYL2	4	NM_017988.6:c.106C>T	NP_060458.3:p.Arg36Ter	Arthrogryposis multiplex congenita	AR
				ERCC8	5	NM_000082.4:c.598_600delinsAA	NP_000073.1:p.Tyr200LysfsTer12	Cockayne syndrome type A	AR
			X	CPS1	5	NM_001875.5:c.2494G>T	NP_001866.2:p.Glu832Ter	Congenital hyperammonemia, type I	AR
				TMPRSS3	5	NM_024022.4:c.1363T>C	NP_076927.1:p.Ter455ArgextTer9	Deafness	AR
42				GUCY2D	4	NM_000180.4:c.1433_1442dup	NP_000171.1:p.Phe482GlyfsTer78	Leber congenital amaurosis	AR
			X	MMAA	5	NM_172250.3:c.433C>T	NP_758454.1:p.Arg145Ter	Vitamin B12-responsive methylmalonic acidemia type cblA	AR
				KIF1C	5	NM_006612.6:c.1667-1G>T		Ataxia, spastic, 2, autosomal recessive	AR
				NUP188	5	NM_015354.3:c.88-2A>G		Sandestig-stefanova syndrome	AR
				PKLR	5	NM_000298.6:c.1456C>T	NP_000289.1:p.Arg486Trp	Pyruvate kinase deficiency of red cells	AR
				METTL23	5	NM_001080510.5:c.434_438del	NP_001073979.3:p.Leu145GlnfsTer12	Mental retardation	AR
				ADAMTS13	5	NM_139025.5:c.1193G>A	NP_620594.1:p.Arg398His	Upshaw-Schulman syndrome	AR
				KIRREL	5	NM_018240.7:c.1718C>T	NP_060710.3:p.Ser573Leu	Nephrotic syndrome	AR
43		X		DPYD	4	NM_000110.4:c.3034G>T	NP_000101.2:p.Glu1012Ter	Dihydropyrimidine dehydrogenase deficiency	AR
				RAD51C	4	NM_058216.3:c.414G>C	NP_478123.1:p.Leu138Phe	Breast-ovarian cancer	AD
				TMPRSS3	4	NM_024022.4:c.413C>A	NP_076927.1:p.Ala138Glu	Deafness	AR
				GJC2	5	NM_020435.4:c.999C>A	NP_065168.2:p.Tyr333Ter	Leukodystrophy, hypomyelinating	AR
				COL9A3	5	NM_001853.4:c.268C>T	NP_001844.3:p.Arg90Ter	Epiphyseal dysplasia, multiple	AD
				ABCC6	5	NM_001171.6:c.2788-1G>A		Generalized arterial calcification of infancy	AR
				SACS	5	NM_014363.6:c.7533del	NP_055178.3:p.Asn2512MetfsTer17	Charlevoix-Saguenay spastic ataxia	AR
44				CGNT2	5	NM_145649.5:c.14G>A	NP_663624.1:p.Trp5Ter	Cataract 13 with adult i	AD
		X		GBA	5	NM_000157.4:c.222_224del	NP_000148.2:p.Thr75del	Lewy body dementia	AD
45				BCHE	4	NM_000055.4:c.635C>T	NP_000046.1:p.Ala212Val	Deficiency of butyrylcholine esterase	AR
				EDAR	5	NM_022336.4:c.126del	NP_071731.1:p.Leu43CysfsTer60	Ectodermal dysplasia	AR
				B3GLCT	5	NM_194318.4:c.482del	NP_919299.3:p.Leu161TyrfsTer8	Peters plus	AR
46				NEK8	4	NM_178170.3:c.1384C>T	NP_835464.1:p.Arg462Ter	renal-hepatic-pancreatic dysplasia	AR
	X			MUTYH	4	NM_012222.3:c.316C>T	NP_036354.1:p.Arg106Trp	Polyposis	AD,AR
				RARS	4	NM_002887.4:c.1367C>T	NP_002878.2:p.Ser456Leu	Leukodystrophy	AR
				TYR	4	NM_000372.5:c.823G>T	NP_000363.1:p.Val275Phe	Albinism	AR
				ABCA4	4	NM_000350.3:c.3499del	NP_000341.2:p.Gln1167ArgfsTer29	Retinitis pigmentosa	AR
				FBP1	5	NM_000507.4:c.960delinsGG	NP_000498.2:p.Ser321ValfsTer13	Fructose biphosphatse deficiency	AR
				THOC6	5	NM_024339.5:c.1003C>T	NP_077315.2:p.Arg335Ter	Beaulieu Boycott sd	AR
				PTHLH	5	NM_198965.2:c.534A>G	NP_945316.1:p.Ter178TrpextTer53	Brachydactyly	AD
		X		CPT2	5	NM_000098.3:c.149C>A	NP_000089.1:p.Pro50His	Carnitine palmitoyltransferase II deficiency	AR
				TECPR2	5	NM_014844.5:c.2664C>G	NP_055659.2:p.Tyr888Ter	SPG49	AR
				LMAN1	5	NM_005570.4:c.1112_1113del	NP_005561.1:p.Ser371Ter	combined deficiency of FV and FVIII	AR
				PROC	5	NM_000312.4:c.1335C>G	NP_000303.1:p.Ile445Met	Thrombophilia	AD,AR
				TIMM22	5	NM_013337.4:c.97G>C	NP_037469.2:p.Val33Leu	Combined oxidative phosphorylation deficiency	AR
47				F7	4	NM_000131.4:c.1061C>T	NP_000122.1:p.Ala354Val	Factor VII deficiency	AR
				PTPRO	5	NM_002848.4:c.1658del	NP_002839.1:p.Pro553LeufsTer3	Nephrotic syndrome type 6	AR
				MAN1B1	5	NM_016219.5:c.620+1G>A		MRAR15	AR
				CNGA1	5	NM_000087.5:c.947C>T	NP_000078.3:p.Ser316Phe	Retinitis pigmentosa	AR
48				CAPN3	4	NM_000070.3:c.2314_2317del	NP_000061.1:p.Asp772AsnfsTer3	Limb-girdle muscular dystrophy	AD
	X			SCN5A	4	NM_000335.5:c.1890+2T>G		cardiopathy	AD
				ABCB11	5	NM_003742.4:c.1826_1827dup	NP_003733.2:p.Ile610GlnfsTer45	familial cholestasis	AR
				LAMA2	5	NM_000426.4:c.6160C>T	NP_000417.3:p.Gln2054Ter	Limb-girdle muscular dystrophy	AR
				NCF1	5	NM_000265.6:c.579G>A	NP_000256.4:p.Trp193Ter	chronic granulomatous disease	AR
				DNMT3A	5	NM_022552.5:c.2478+1dup		Tatton-Brown	AD
				FLG	5	NM_002016.2:c.1501C>T	NP_002007.1:p.Arg501Ter	ichthyosis vulgaris	AD
49				SLC24A1	4	NM_004727.3:c.1132_1133del	NP_004718.1:p.Ser378AsnfsTer45	night blindness	AR
	X			BRCA2	4	NM_000059.4:c.5241_5242del	NP_000050.3:p.Asn1747LysfsTer7	Breast cancer	AR
				VPS13B	5	NM_017890.5:c.11314C>T	NP_060360.3:p.Gln3772Ter	Cohen	AR
				MYO15A	5	NM_016239.4:c.3385C>T	NP_057323.3:p.Arg1129Ter	Deafness	AR
				RIMS2	5	NM_014677.5:c.1549_1553del	NP_055492.3:p.Ile517CysfsTer28	Cone rode synaptic disorder	AR
				PEDP	5	NM_000285.4:c.441+1G>A		Prolipase deficiency	AR

				TRNT1	5	NM_182916.3:c.33_46del	NP_886552.3:p.Leu13GlufsTer2	Sideroblastic anemia with B-cell immunodeficiency, periodic fevers, and d	AR
				TMEM67	5	NM_153704.6:c.622A>T	NP_714915.3:p.Arg208Ter	Meckel syndrome	AR
50				RECQL4	4	NM_004260.4:c.1397C>T	NP_004251.4:p.Pro466Leu	Baller Gerold	AR
				GFM1	4	NM_001308164.2:c.729G>A	NP_001295093.1:p.Trp243Ter	Combined owidative phosphorylation deficiency	AR
				C9	4	NM_001737.5:c.1630C>T	NP_001728.1:p.Gln544Ter	C9 deficiency	AR
				SNIP1	4	NM_024700.4:c.331C>T	NP_078976.2:p.Arg111Cys	Psychomotor retardation, epilepsy, and craniofacial dysmorphism	AR
51				CPAMD8	4	NM_015692.5:c.5275G>T	NP_056507.3:p.Glu1759Ter	dysgénésie antérieure	AR
				C2	4	NM_000063.6:c.841_849+19del		Complement component 2 deficiency	AR
52				NLRP12	4	NM_144687.4:c.3088C>T	NP_653288.1:p.Arg1030Ter	Familial cold autoinflammatory syndrome	AD
				PNPT1	4	NM_033109.5:c.2213G>A	NP_149100.2:p.Arg738His	Combined oxidative phosphorylation deficiency	AR
				PDZD7	5	NM_024895.5:c.82C>T	NP_079171.1:p.Arg28Ter	Usher syndrome	AR
		X		MEFV	5	NM_000243.3:c.730G>T	NP_000234.1:p.Glu244Ter	Familial Mediterranean fever	AR
				MEGF8	5	NM_001410.3:c.4629+2T>C		Carpenter	AR
	X			TTN	5	NM_003319.4:c.29453-1G>C		Familial hypertrophic cardiomyopathy	AD
				TOP3A	5	NM_004618.5:c.194C>A	NP_004609.1:p.Ser65Ter	Microcephaly, growth restriction, and increased sister chromatid exchange	AR
				SOX2	5	NM_003106.4:c.389G>C	NP_003097.1:p.Gly130Ala	Anophthalmia/microphthalmia-esophageal atresia syndrome	AD
				ZMPSTE24	5	NM_005857.5:c.1085dup	NP_005848.2:p.Leu362PhefsTer19	Lethal tight skin contracture syndrome	AR
53				UBE3B	4	NM_001270449.1:c.711G>A	NP_001257378.1:p.Trp237Ter	Kaufman oculocerebrofacial syndrome	AR
				AP4B1	4	NM_001253853.1:c.2T>C	NP_001240782.1:p.Met1?	Spastic paraplegia	AR
				C2	4	NM_000063.4:c.841_849+19del		Complement component 2 deficiency	AR
				FGD1	4	NM_004463.2:c.527dup	NP_004454.2:p.Leu177ThrfsTer40	Aarskog syndrome	XLR
				DIAPH1	5	NM_005219.4:c.3149-2_3149-1delinsTTAT		Seizures, cortical blindness, and microcephaly syndrome	AD,AR
				SLC13A5	5	NM_177550.3:c.1022G>A	NP_808218.1:p.Trp341Ter	Epileptic encephalopathy	AR
				TSHR	5	NM_000369.2:c.1170T>G	NP_000360.2:p.Cys390Trp	Hyperthyroidism	AD,AR
54	X			BRCA2	4	NM_000059.3:c.9934del	NP_000050.2:p.Ile3312Ter	Breast-ovarian cancer	AD
				C2CD3	4	NM_015531.5:c.5761C>T	NP_056346.3:p.Gln1921Ter	Orofaciodigital syndrome xiv	AR
		X		GJB2	5	NM_004004.5:c.167del	NP_003995.2:p.Leu56ArgfsTer26	Deafness	AR
				OBSL1	5	NM_015311.2:c.621C>G	NP_056126.1:p.Tyr207Ter	Three M syndrome	AR
				LTBP2	5	NM_000428.2:c.4454-2A>G		Weill-Marchesani syndrome	AR
				TRIM32	5	NM_012210.3:c.2T>C	NP_036342.2:p.Met1?	Bardet-Biedl syndrome	AR
				NPHS2	5	NM_014625.3:c.413G>A	NP_055440.1:p.Arg138Gln	Idiopathic nephrotic syndrome	AR
				OCA2	5	NM_000275.2:c.1327G>A	NP_000266.2:p.Val443Ile	Tyrosinase-positive oculocutaneous albinism	AR
				DRD4	5	NM_000797.3:c.235_247del	NP_000788.2:p.Ala79SerfsTer21	Autonomic nervous system dysfunction	AD
55				FLG	4	NM_002016.1:c.10252del	NP_002007.1:p.Ala3418HisfsTer37	Ichthyosis vulgaris	AD
				VAMP2	4	NM_014232.2:c.87del	NP_055047.2:p.Asn29LysfsTer18	Neurodevelopmental disorder with hypotonia and autistic features with or	AD
				PRG4	4	NM_005807.3:c.531_535del	NP_005798.2:p.Thr178SerfsTer12	Camptodactyly arthropathy coxa vara pericarditis syndrome	AR
				FAN1	5	NM_014967.4:c.2489-1G>A		Interstitial nephritis, karyomegalic	AR
				CYB5R3	5	NM_000398.6:c.316G>A	NP_000389.1:p.Val106Met	Deficiency of cytochrome-b5 reductase	AR
56		X	X	HBB	4	NM_000518.4:c.92+1G>A		Beta-thalassemia	AR
				RPGRIP1	4	NM_020366.3:c.1484_1496del	NP_065099.3:p.Asn495ArgfsTer7	Leber congenital amaurosis	AR
57				GJB4	4	NM_153212.2:c.65G>A	NP_694944.1:p.Arg22His	Deafness	AR
		X		PYGM	5	NM_005609.2:c.148C>T	NP_005600.1:p.Arg50Ter	Glycogen storage disease	AR
				ABCA1	5	NM_005502.3:c.5383-2_5383-1delinsTTTAT		Tangier disease	AR
58	X			MYL2	4	NM_000432.3:c.431del	NP_000423.2:p.Pro144LeufsTer3	Familial hypertrophic cardiomyopathy	AD
				CUL7	5	NM_014780.4:c.4369_4370del	NP_055595.2:p.Gln1457ValfsTer96	Yakut short stature syndrome	AR
				SLC3A1	5	NM_000341.3:c.1400T>C	NP_000332.2:p.Met467Thr	Cystinuria	AR
				EYS	5	NM_001142800.1:c.2135_2136dup	NP_001136272.1:p.Val713LysfsTer17	Retinitis pigmentosa	AR
				NARS2	5	NM_024678.5:c.1209del	NP_078954.4:p.Phe403LeufsTer13	Combined oxidative phosphorylation deficiency	AR
				GORAB	5	NM_152281.2:c.975_983delinsG	NP_689494.2:p.Arg326SerfsTer21	Geroderma osteodysplastica	AR
				ZNF687	5	NM_020832.1:c.2810C>G	NP_065883.1:p.Pro937Arg	Paget disease of bone	AD
				PADI3	5	NM_016233.2:c.881C>T	NP_057317.2:p.Ala294Val	Uncombable hair syndrome	AR
59				PDE11A	5	NM_016953.3:c.1737dup	NP_058649.3:p.Val580CysfsTer12	Pigmented nodular adrenocortical disease,	AD
				GALNT3	5	NM_004482.3:c.1209del	NP_004473.2:p.Gln404SerfsTer28	Tumoral calcinosis, hyperphosphatemic, familial	AR
60				CHEK2	4	NM_007194.3:c.433C>T	NP_009125.1:p.Arg145Trp	Li-Fraumeni syndrome	AD
				BCHE	4	NM_000055.2:c.635C>T	NP_000046.1:p.Ala212Val	Deficiency of butyrylcholine esterase	AR
				DCC	5	NM_005215.3:c.2165-2A>G		Mirror movements	AD,AR
				DNA2	5	NM_001080449.2:c.971_972dup	NP_001073918.2:p.Arg325GlufsTer26	Seckel syndrome	AD,AR

				WARS2	5	NM_015836.3:c.702del	NP_056651.1:p.Lys234AsnfsTer7	Neurodevelopmental disorder, mitochondrial, with abnormal movements a	AR
				MPO	5	NM_000250.1:c.2031-2A>C		Myeloperoxidase deficiency	AR
61				PGAM2	4	NM_000290.3:c.533del	NP_000281.2:p.Gly178AlafsTer31	Glycogen storage disease	AR
				MVK	4	NM_000431.2:c.794T>G	NP_000422.1:p.Leu265Arg	Mevalonic aciduria	AR
				GLI1	4	NM_005269.2:c.2709_2710del	NP_005260.1:p.Gln903HisfsTer34	Polydactyly	AR
				DSG1	4	NM_001942.2:c.3106C>T	NP_001933.2:p.Arg1036Ter	Erythroderma, congenital, with palmoplantar keratoderma, hypotrichosis, a	AD,AR
				TEX11	4	NM_031276.2:c.466A>G	NP_112566.2:p.Met156Val	Spermatogenic failure	XLR
				TTC21B	5	NM_024753.4:c.626C>T	NP_079029.3:p.Pro209Leu	Nephronophthisis	AR
				ATP6V0A4	5	NM_020632.2:c.322C>T	NP_065683.2:p.Gln108Ter	Renal tubular acidosis	AR
				DNAH1	5	NM_015512.4:c.11706C>G	NP_056327.4:p.Tyr3902Ter	Spermatogenic failure 18	AR
				AICDA	5	NM_020661.3:c.428-4_428-1delinsTTAAT		Immunodeficiency with hyper IgM	AR
62				WDR73	4	NM_032856.2:c.1132del	NP_116245.2:p.Arg378AlafsTer25	Galloway-Mowat syndrome	AR
				DBH	5	NM_000787.3:c.339+2T>C		Dopamine beta hydroxylase deficiency	AR
			X	GCDH	5	NM_000159.3:c.679C>T	NP_000150.1:p.Arg227Trp	Glutaric aciduria	AR
		X	X	PAH	5	NM_000277.1:c.1140C>T	NP_000268.1:p.Thr380Met	phenylketonuria	AR
63				EXOSC3	4	NM_016042.4:c.395A>C	NP_057126.2:p.Asp132Ala	Pontocerebellar hypoplasia,	AR
64				SOX18	4	NM_018419.2:c.84dup	NP_060889.1:p.Ala29ArgfsTer3	Hypotrichosis-lymphedema-telangiectasia	AD
65			X	GCDH	4	NM_000159.3:c.852+1G>A		Glutaric aciduria	AR
66			X	IVD	4	NM_002225.5:c.932C>T	NP_002216.3:p.Ala311Val	Isovaleryl-CoA dehydrogenase deficiency	AR
				GP1BA	4	NM_000173.7:c.586C>T	NP_000164.5:p.Gln196Ter	Bernard Soulier syndrome	AD
				CC2D2A	5	NM_001080522.2:c.2848C>T	NP_001073991.2:p.Arg950Ter	Meckel syndrome	AR
				TTC21A	5	NM_145755.2:c.102dup	NP_665698.2:p.Phe35IlefsTer4	Spermatogenic failure 37	AR
				BBS10	5	NM_024685.4:c.271dup	NP_078961.3:p.Cys91LeufsTer5	Bardet-Biedl syndrome	AR
				NPHS1	5	NM_004646.3:c.1099C>T	NP_004637.1:p.Arg367Cys	Finnish congenital nephrotic syndrome	AR
		X	X	CFTR	5	NM_000492.4:c.1521_1523del	NP_000483.3:p.Phe508del	Cystic fibrosis	AR
67			X	HMGCL	4	NM_000191.3:c.853del	NP_000182.2:p.Leu285Ter	Deficiency of hydroxymethylglutaryl-CoA lyase	AR
			X	GAMT	4	NM_000156.6:c.328-1G>A		Deficiency of guanidinoacetate methyltransferase	AR
	X			FBN1	4	NM_000138.4:c.4192G>C	NP_000129.3:p.Asp1398His	Marfan syndrome	AD
				DUOX2	4	NM_014080.4:c.2895_2898del	NP_054799.4:p.Phe966SerfsTer29	Thyroid dyshormonogenesis	AR
				CEP290	5	NM_025114.4:c.180+1G>A		Meckel syndrome	AR
		X	X	PMM2	5	NM_000303.3:c.422G>A	NP_000294.1:p.Arg141His	Carbohydrate-deficient glycoprotein syndrome	AR
				ABCG8	5	NM_022437.3:c.361C>T	NP_071882.1:p.Arg121Ter	Sitosterolemia	AR
				TRMT1	5	NM_017722.4:c.1704-1G>C		Intellectual developmental disorder,	AR
				POLH	5	NM_006502.3:c.2074A>G	NP_006493.1:p.Thr692Ala	Xeroderma pigmentosum	AR
				PADI3	5	NM_016233.2:c.881C>T	NP_057317.2:p.Ala294Val	Uncombable hair syndrome	AR
				PHYKPL	5	NM_153373.4:c.1310A>T	NP_699204.1:p.Glu437Val	Phosphohydroxylysinuria	AR
68				RPGRIP1L	4	NM_015272.5:c.2200C>T	NP_056087.2:p.Arg734Ter	Joubert syndrome	AR
		X	X	PAH	5	NM_000277.3:c.727C>T	NP_000268.1:p.Arg243Ter	Phenylketonuria	AR
				AGXT	5	NM_000030.3:c.731T>C	NP_000021.1:p.Ile244Thr	Primary hyperoxaluria	AR
		X		MEFV	5	NM_000243.2:c.2082G>A	NP_000234.1:p.Met694Ile	Familial Mediterranean fever	AR
				PCK2	5	NM_004563.4:c.577C>T	NP_004554.3:p.Arg193Ter	Phosphoenolpyruvate carboxykinase deficiency	AD,AR
				TIMM22	5	NM_013337.4:c.97G>C	NP_037469.2:p.Val33Leu	Combined oxidative phosphorylation deficiency	AR
69				IFNGR2	4	NM_005534.3:c.73+2T>G		Immunodeficiency	AR
		X	X	CFTR	4	NM_000492.4:c.1392G>T	NP_000483.3:p.Lys464Asn	Cystic fibrosis	AR
	X	X		GAA	5	NM_000152.5:c.1802C>A	NP_000143.2:p.Ser601Ter	Glycogen storage disease,	AR
				NEB	5	NM_004543.5:c.16371_16372dup	NP_004534.3:p.Thr5458IlefsTer54	Nemaline myopathy	AR
				DRC1	5	NM_145038.5:c.352C>T	NP_659475.2:p.Gln118Ter	Ciliary dyskinesia, primary	AR
				SBDS	5	NM_016038.4:c.258+2T>C		Shwachman-Diamond syndrome	AR
70				SI	4	NM_001041.4:c.3186_3187del	NP_001032.2:p.Tyr1063Ter	Sucrase-isomaltase deficiency	AR
				TMC1	5	NM_138691.2:c.1677G>A	NP_619636.2:p.Trp559Ter	Deafness	AD,AR
				TEX15	5	NM_001350162.2:c.4148del	NP_001337091.1:p.Ala1383GlufsTer5	Spermatogenic failure	AR
				GSS	5	NM_000178.4:c.656A>G	NP_000169.1:p.Asp219Gly	Gluthathione synthetase deficiency	AR
				FLG	5	NM_002016.2:c.7339C>T	NP_002007.1:p.Arg2447Ter	Ichthyosis vulgaris	AD
71	X			DSP	5	NM_004415.4:c.1590C>A	NP_004406.2:p.Tyr530Ter	Cardiomyopathy dilated with woolly hair and keratoderma	AD
72				ASPM	5	NM_018136.5:c.7782_7783del	NP_060606.3:p.Lys2595SerfsTer6	Primary autosomal recessive microcephaly	AR
				TMEM231	5	NM_001077416.2:c.248C>A	NP_001070884.2:p.Ser83Ter	Meckel syndrome	AR
				CEP78	5	NM_032171.3:c.1393G>T	NP_115547.1:p.Glu465Ter	Cone-rod dystrophy and hearing loss	AR

				ACSF3	5	NM_174917.5:c.155_161del	NP_777577.2:p.Ala52GlyfsTer64	Combined malonic and methylmalonic aciduria	AR
		X	X	PAH	5	NM_000277.3:c.898G>T	NP_000268.1:p.Ala300Ser	phenylketonuria	AR
				FLG	5	NM_002016.2:c.1501C>T	NP_002007.1:p.Arg501Ter	Ichthyosis vulgaris	AD
73				SLC26A4	4	NM_000441.2:c.-3-2A>G		Pendred syndrome	AR
				GOSR2	4	NM_001012511.2:c.580del	NP_001012529.1:p.Ile194TyrfsTer51	Epilepsy, progressive myoclonic	AR
				SACS	5	NM_014363.6:c.7205_7206del	NP_055178.3:p.Leu2402ArgfsTer6	Charlevoix-Saguenay spastic ataxia	AR
				ADCY10	5	NM_018417.6:c.3907dup	NP_060887.2:p.Val1303GlyfsTer15	Familial idiopathic hypercalciuria	AD
				SPEG	5	NM_005876.5:c.8539C>T	NP_005867.3:p.Arg2847Ter	Myopathy, centronuclear	AR
				ARV1	5	NM_022786.3:c.*4+1G>A		Epileptic encephalopathy, early infantile	AR
				FLG	5	NM_002016.2:c.1501C>T	NP_002007.1:p.Arg501Ter	Ichthyosis vulgaris	AD
74				CD36	4	NM_000072.3:c.584_586delinsTT	NP_000063.2:p.Thr195IlefsTer27	Platelet glycoprotein IV deficiency	AR
				DYSF	5	NM_003494.4:c.2643+1G>A		Limb-girdle muscular dystrophy	AR
				C5	5	NM_001735.3:c.55C>T	NP_001726.2:p.Gln19Ter	Eculizumab, poor response to	AD,AR
75				CD36	5	NM_000072.3:c.1110_1119del	NP_000063.2:p.Tyr370Ter	Platelet glycoprotein IV deficiency	AD,AR
				COL6A3	5	NM_004369.4:c.865G>T	NP_004360.2:p.Glu289Ter	Bethlem myopathy	AD,AR
76				STRC	4	NM_153700.2:c.4012C>T	NP_714544.1:p.Arg1338Ter	Deafness	AR
				SYCE1	5	NM_130784.3:c.163+1G>A		Spermatogenic/Premature ovarian failure	AR
				FLG	5	NM_002016.2:c.1501C>T	NP_002007.1:p.Arg501Ter	Ichthyosis vulgaris	AD
				VWF	5	NM_000552.4:c.2561G>A	NP_000543.2:p.Arg854Gln	von Willebrand disease	AD
77		X		PYGM	4	NM_005609.4:c.2262del	NP_005600.1:p.Lys754AsnfsTer49	Glycogen storage disease	AR
				SLC7A7	4	NM_001126105.2:c.1273T>C	NP_001119577.1:p.Cys425Arg	Lysinuric protein intolerance	AR
				FANCE	5	NM_021922.3:c.265C>T	NP_068741.1:p.Arg89Ter	Fanconi anemia	AR
				PLCE1	5	NM_016341.4:c.136C>T	NP_057425.3:p.Arg46Ter	Nephrotic syndrome	AR
78				DNAH5	4	NM_001369.2:c.5503C>T	NP_001360.1:p.Gln1835Ter	Ciliary dyskinesia, primary	AR
				MRPS22	4	NM_020191.4:c.40_41insA	NP_064576.1:p.Leu14TyrfsTer49	Combined oxidative phosphorylation deficiency	AR
				BPTF	5	NM_004459.7:c.255dup	NP_004450.3:p.Ser86GlnfsTer43	Neurodevelopmental disorder with dysmorphic facies and distal limb anon	AD
				CEP55	5	NM_018131.5:c.841C>T	NP_060601.4:p.Gln281Ter	Multinucleated neurons, anhydramnios, renal dysplasia, cerebellar hypopla	AR
				CLCN2	5	NM_004366.6:c.2572C>T	NP_004357.3:p.Arg858Ter	Leukoencephalopathy with ataxia	AD,AR
				DYSF	5	NM_003494.4:c.4200dup	NP_003485.1:p.Ile1401HisfsTer8	Limb-girdle muscular dystrophy	AR
79				ADA	4	NM_000022.2:c.311C>T	NP_000013.2:p.Pro104Leu	Severe combined immunodeficiency	AR
				SCARF2	4	NM_153334.6:c.2297_2298delinsTGC	NP_699165.3:p.Ala766ValfsTer10	Van den Ende-Gupta syndrome	AR
				CNTN2	5	NM_005076.4:c.2126-1G>A		Epilepsy, familial adult myoclonic	AR
				CD36	5	NM_000072.3:c.157_158delinsG	NP_000063.2:p.Asn53ValfsTer24	Platelet glycoprotein IV deficiency	AR
				PADI3	5	NM_016233.2:c.856A>G	NP_057317.2:p.Thr286Ala	Uncombable hair syndrome	AR
80				CEP152	4	NM_014985.3:c.2034T>G	NP_055800.2:p.Tyr678Ter	Primary autosomal recessive microcephaly 9	AR
				FSIP2	4	NM_173651.2:c.15694_15695del	NP_775922.2:p.Lys5232ValfsTer5	Spermatogenic failure	AR
				IMPG1	4	NM_001563.2:c.672del	NP_001554.2:p.Glu225LysfsTer19	Macular dystrophy	AD
				BRAT1	4	NM_152743.3:c.2068G>T	NP_689956.2:p.Glu690Ter	Rigidity and multifocal seizure syndrome	AR
				CYP4V2	5	NM_207352.3:c.332T>C	NP_997235.3:p.Ile111Thr	Bietti crystalline corneoretinal dystrophy	AR
				MCCC2	5	NM_022132.4:c.1015G>A	NP_071415.1:p.Val339Met	3-methylcrotonyl CoA carboxylase 2 deficiency	AR
				KDM5B	5	NM_006618.3:c.3261_3264+17delinsGAGCTG		Mental retardation	AR
				COG6	5	NM_020751.2:c.1892dup	NP_065802.1:p.Met632AspfsTer6	Congenital disorder of glycosylation type 2L	AR
				UGT1A1	5	NM_000463.2:c.1070A>G	NP_000454.1:p.Gln357Arg	Crigler-Najjar syndrome	AR
				SMN2	5	NM_017411.3:c.859G>C	NP_059107.1:p.Gly287Arg	Kugelberg-Welander disease	AR
81				HBA1	5	NM_000558.4:c.358C>T	NP_000549.1:p.Pro120Ser	alpha thalassemia	AD
				CUL7	5	NM_014780.4:c.3136del	NP_055595.2:p.Leu1046TrpfsTer95	Yakut short stature syndrome	AR
				CEP55	5	NM_018131.4:c.190C>T	NP_060601.3:p.Arg64Ter	Multinucleated neurons, anhydramnios, renal dysplasia, cerebellar hypopla	AR
				NR2E3	5	NM_014249.3:c.932G>A	NP_055064.1:p.Arg311Gln	Retinitis pigmentosa	AR
82				OTOG	4	NM_001277269.1:c.603_604del	NP_001264198.1:p.Ser202ThrfsTer15	Deafness	AR
	X			HNF1A	4	XM_005253932.1:c.1060_1062delinsTCATTCAT	XP_005253989.1:p.Thr354SerfsTer24	Maturity-onset diabetes of the young	AD
				SLC12A3	4	NM_000339.2:c.37G>C	NP_000330.2:p.Ala13Pro	Familial hypokalemia-hypomagnesemia	AR
				TACR3	5	NM_001059.2:c.824G>A	NP_001050.1:p.Trp275Ter	Hypogonadotropic hypogonadism 11 with or without anosmia	AR
				ERCC4	5	NM_005236.2:c.1407_1414del	NP_005227.1:p.Lys470ProfsTer30	Xeroderma pigmentosum	AR
				EARS2	5	NM_001083614.1:c.322C>T	NP_001077083.1:p.Arg108Trp	Combined oxidative phosphorylation deficiency 12	AR
				DUOX2	5	NM_014080.4:c.2895_2898del	NP_054799.4:p.Phe966SerfsTer29	Thyroid dyshormonogenesis	AR

83				PLEC	4	NM_201384.2:c.7C>T	NP_958786.1:p.Gln3Ter	Limb-girdle muscular dystrophy,	AR
				TG	5	NM_003235.4:c.5184C>A	NP_003226.4:p.Cys1728Ter	Iodotyrosyl coupling defect	AR
				ABCA4	5	NM_000350.2:c.3113C>T	NP_000341.2:p.Ala1038Val	Retinitis pigmentosa	AR
84				SERPINA1	4	NM_000295.5:c.187C>T	NP_000286.3:p.Arg63Cys	Alpha-1-antitrypsin deficiency	AR
				ABCC6	4	NM_001171.5:c.3143_3145del	NP_001162.4:p.Phe1048del	Generalized arterial calcification of infancy	AR
			X	CYP27A1	5	NM_000784.4:c.844+1G>A		Cholestanol storage disease	AR
				DNAH5	5	NM_001369.2:c.5710-1del		Ciliary dyskinesia, primary	AR
				C5orf42	5	NM_023073.3:c.7121C>G	NP_075561.3:p.Ser2374Ter	Joubert syndrome	AR
85				TCIRG1	4	NM_006019.4:c.979C>T	NP_006010.2:p.Arg327Ter	Osteopetrosis autosomal recessive	AR
				TSEN54	5	NM_207346.3:c.919G>T	NP_997229.2:p.Ala307Ser	Pontocerebellar hypoplasia	AR
		X		CPT2	5	NM_000098.3:c.149C>A	NP_000089.1:p.Pro50His	Carnitine palmitoyltransferase II deficiency	AR
				MPZL2	5	NM_005797.4:c.72del	NP_005788.1:p.Ile24MetfsTer22	Deafness	AR
				KIAA0586	5	NM_014749.5:c.392del	NP_055564.3:p.Arg131LysfsTer4	Joubert syndrome	AR
86				FLG	4	NM_002016.2:c.9253G>T	NP_002007.1:p.Gly3085Ter	Ichthyosis vulgaris	AD
		X	X	HBB	4	NM_000518.5:c.92G>C	NP_000509.1:p.Arg31Thr	beta Thalassemia	AR
	X			TTN	5	NM_133378.4:c.20362C>T	NP_596869.4:p.Gln6788Ter	Familial hypertrophic cardiomyopathy	AD
				MYO7A	5	NM_000260.4:c.3904del	NP_000251.3:p.Tyr1302ThrfsTer97	Usher syndrome	AR
				G6PD	5	NM_000402.4:c.961G>A	NP_000393.4:p.Val321Met	Glucose 6 phosphate dehydrogenase deficiency	XLR
				FANCI	5	NM_018193.3:c.756-1G>C		Fanconi anemia	AR
87				GRM6	4	NM_000843.4:c.-16-2A>C		Congenital stationary night blindness	AR
				EXOSC3	5	NM_016042.4:c.395A>C	NP_057126.2:p.Asp132Ala	Pontocerebellar hypoplasia,	AR
				PIK3R2	5	NM_005027.4:c.815+2T>G		MEGALENCEPHALY-POLYMICROGYRIA-POLYDACTYLY-HYDROCEPHALUS	AD
88				SERPINC1	4	NM_000488.3:c.391C>T	NP_000479.1:p.Leu131Phe	Antithrombin III deficiency	AD,AR
				ITGA2B	5	NM_000419.5:c.1544+1G>A		Glanzmann thrombasthenia	AD,AR
				DBH	5	NM_000787.4:c.339+2T>C		Dopamine beta hydroxylase deficiency	AR
				ADPRHL2	5	NM_017825.3:c.472del	NP_060295.1:p.Ala158LeufsTer23	Neurodegeneration, childhood-onset, stress-induced, with variable ataxia a	AD
			X	HMGCS2	5	NM_005518.4:c.105-1G>A		mitochondrial 3-hydroxy-3-methylglutaryl-CoA synthase deficiency	AR
89				TMPRSS3	4	NM_024022.3:c.1276G>A	NP_076927.1:p.Ala426Thr	Deafness	AR
				TARDBP	4	NM_007375.3:c.881G>T	NP_031401.1:p.Gly294Val		AD,AR
				TTR	5	NM_000371.3:c.424G>A	NP_000362.1:p.Val142Ile	Amyloidogenic transthyretin amyloidosis	AD
				PIEZO1	5	NM_001142864.4:c.4293_4312del	NP_001136336.2:p.Pro1433GlyfsTer85	Xerocytosis	AD,AR
90				DSG4	4	NM_177986.5:c.2219dup	NP_817123.1:p.Ala741SerfsTer31	Hypotrichosis 6	AR
				ADAMTSL4	5	NM_019032.5:c.963dup	NP_061905.2:p.Thr322AspfsTer10	Ectopia lentis	AR
				DNAH9	5	NM_001372.4:c.10367del	NP_001363.2:p.Leu3456ProfsTer43	Ciliary dyskinesia, primary	AR
				CD36	5	NM_000072.3:c.2T>C	NP_000063.2:p.Met1?	Platelet glycoprotein IV deficiency	AD,AR
				VWF	5	NM_000552.4:c.5347T>G	NP_000543.2:p.Ser1783Ala	von Willebrand disease	AD
91				RTTN	5	NM_173630.4:c.3589_3590del	NP_775901.3:p.Asp1197Ter	Microcephaly, short stature, and polymicrogyria with or without seizures	AR
		X		CPT2	5	NM_000098.3:c.338C>T	NP_000089.1:p.Ser113Leu	Carnitine palmitoyltransferase II deficiency	AR
				VPS33B	5	NM_018668.5:c.1567C>T	NP_061138.3:p.Arg523Ter	Arthrogryposis,	AR
				FLG	5	NM_002016.2:c.1501C>T	NP_002007.1:p.Arg501Ter	Ichthyosis vulgaris,	AD
92				SPG7	5	NM_003119.4:c.1169T>C	NP_003110.1:p.Val390Ala	Hereditary spastic paraplegia	AD,AR
				FANCA	5	NM_000135.4:c.1115_1118del	NP_000126.2:p.Val372AlafsTer42	Fanconi anemia	AR
93				EIF2B2	4	NM_014239.4:c.599G>T	NP_055054.1:p.Gly200Val	Leukoencephalopathy with vanishing white matter	AR
				EIF2B4	4	NM_015636.4:c.725C>T	NP_056451.3:p.Pro242Leu	Leukoencephalopathy with vanishing white matter	AR
				TEX15	4	NM_001350162.2:c.2574_2577del	NP_001337091.1:p.Thr859IlefsTer16	Spermatogenic failure	AR
				BCHE	4	NM_000055.4:c.812C>T	NP_000046.1:p.Thr271Met	Deficiency of butyrylcholine esterase	AR
				CNGA3	5	NM_001298.3:c.829C>T	NP_001289.1:p.Arg277Cys	Achromatopsia	AR
				CFAP58	5	NM_001008723.2:c.2182C>T	NP_001008723.1:p.Gln728Ter	Spermatogenic	AR
				GFM1	5	NM_024996.7:c.1027_1031del	NP_079272.4:p.Met343LeufsTer3	Combined oxidative phosphorylation deficiency 1	AR
				RSPO4	5	NM_001029871.4:c.79+1G>A		Anonychia congenita	AR
			X	CYP27A1	5	NM_000784.4:c.1213C>T	NP_000775.1:p.Arg405Trp	Cholestanol storage disease	AR
94				SLC25A20	4	NM_000387.6:c.397C>T	NP_000378.1:p.Arg133Trp	Carnitine acylcarnitine translocase deficiency	AR
				GDAP1	5	NM_018972.4:c.373C>T	NP_061845.2:p.Arg125Ter	Charcot-Marie-Tooth disease	AR
				HBA1	5	NM_000558.5:c.358C>T	NP_000549.1:p.Pro120Ser	alpha Thalassemia	AD

			POLR3A	5	NM_007055.4:c.473dup	NP_008986.2:p.His158GlnfsTer6	Hypomyelinating leukodystrophy	AR
95			RECQL4	4	NM_004260.3:c.1397C>T	NP_004251.3:p.Pro466Leu	Baller Gerold	AR
			IMPG1	4	NM_001563.4:c.1262del	NP_001554.2:p.Thr421LysfsTer39	Macular dystrophy	AD
		X	DLD	5	NM_000108.5:c.685G>T	NP_000099.2:p.Gly229Cys	Deficiency of cytochrome-b5 reductase	AR
			CAPN3	5	NM_000070.3:c.1466G>A	NP_000061.1:p.Arg489Gln	Limb-girdle muscular dystrophy	AR
			PDE6A	5	NM_000440.3:c.2072_2073del	NP_000431.2:p.Tyr691Ter	Retinitis pigmentosa	AR
			UPB1	5	NM_016327.3:c.105-2A>G		Deficiency of beta-ureidopropionase	AR
			RBBP8	5	NM_002894.3:c.2028+1G>T		Seckel syndrome	AR
			NANOS1	5	NM_199461.4:c.517_519del	NP_955631.1:p.Ala173del	Spermatogenic failure	AD
96			SLC7A9	5	NM_014270.5:c.544G>A	NP_055085.1:p.Ala182Thr	Cystinuria	AR
			ANKH	5	NM_054027.6:c.1468_1470del	NP_473368.1:p.Glu490del	Craniometaphyseal dysplasia	AD
97	X		TTN	5	NM_003319.4:c.30091_30092insACCTTCAG	NP_003310.4:p.Ala10031AspfsTer17	Familial hypertrophic cardiomyopathy	AD
			NPHS1	5	NM_004646.3:c.1315+1G>A		Finnish congenital nephrotic syndrome	AR
			APOE	5	NM_000041.4:c.940A>C	NP_000032.1:p.Ser314Arg	Lipoprotein glomerulopathy	
98			TMEM5	5	NM_014254.3:c.429-2A>G		Muscular dystrophy-dystroglycanopathy	AR
			SYNE4	5	NM_001039876.3:c.677G>A	NP_001034965.1:p.Trp226Ter	Deafness	AR
99			PCK2	4	NM_004563.4:c.708delinsAA	NP_004554.3:p.Ile237AsnfsTer40	Phosphoenolpyruvate carboxykinase deficiency	AD,AR
			FANCM	5	NM_020937.4:c.5791C>T	NP_065988.1:p.Arg1931Ter	Fanconi anemia	AR
			PARK2	5	NM_004562.3:c.823C>T	NP_004553.2:p.Arg275Trp	Parkinson disease	AR
			CRB1	5	NM_201253.3:c.498_506del	NP_957705.1:p.Ile167_Gly169del	Leber congenital amaurosis	AR
100			KIF14	4	NM_014875.3:c.131dup	NP_055690.1:p.Met44IlefsTer5	Meckel syndrome	AR
			FANCA	5	NM_000135.4:c.1115_1118del	NP_000126.2:p.Val372AlafsTer42	Fanconi anemia	AR
			NCKAP1L	5	NM_005337.5:c.1076C>T	NP_005328.2:p.Pro359Leu	Immunodeficiency	AR
			DLG4	5	NM_001365.4:c.280-2T>C		Intellectual developmental disorder	AD
		X	MMACHC	5	NM_015506.3:c.271dup	NP_056321.2:p.Arg91LysfsTer14	Cobalamin C	AR
			PADI3	5	NM_016233.2:c.881C>T	NP_057317.2:p.Ala294Val	Uncombable hair syndrome	AR
			HOXB13	5	NM_006361.6:c.251G>A	NP_006352.2:p.Gly84Glu	Prostate cancer	AD
